# To the Medical Practitioners of Kentucky

**Published:** 1853-03

**Authors:** 


TO THE MEDICAL PRACTITIONERS OF KENTUCKY.

At the last meeting of the State Medical Society of Kentucky, the undersigned was appointed Chairman of the Committee on Surgery; and he requests the medical gentlemen in different parts of the State to communicate to him an account of every case, or professional observations of special interest, that have recently occurred to them in the surgical departments of their practice.

He would particularly request precise and circumstantial replies to the following inquiry, from any who can make them affirmatively, viz:

Have you ever known any — and if so, how many — instances of death from uncomplicated disease of the ovaria, and what was the age of the patient or patients at the time of death?

Communications in response to this call should be forwarded, if possible, by the first of September next.

JOSHUA B. FLINT.

Louisville, March 1st, 1853.

